# Cellular Plasticity in Prostate Cancer Bone Metastasis

**DOI:** 10.1155/2015/651580

**Published:** 2015-06-03

**Authors:** Dima Y. Jadaan, Mutaz M. Jadaan, John P. McCabe

**Affiliations:** ^1^University College Dublin, Belfield, Dublin, Ireland; ^2^Department of Orthopaedic Surgery, Galway University Hospitals, Galway, Ireland

## Abstract

*Purpose.* Experimental data suggest that tumour cells can reversibly transition between epithelial and mesenchymal states (EMT and MET), a phenomenon known as cellular plasticity. The aim of this review was to appraise the clinical evidence for the role of cellular plasticity in prostate cancer (PC) bone metastasis.* Methods.* An electronic search was performed using PubMed for studies that have examined the differential expression of epithelial, mesenchymal, and stem cell markers in human PC bone metastasis tissues.* Results.* The review included nineteen studies. More than 60% of the studies used ≤20 bone metastasis samples, and there were several sources of heterogeneity between studies. Overall, most stem cell markers analysed, except for CXCR4, were positively expressed in bone metastasis tissues, while the expression of EMT and MET markers was heterogeneous between and within samples. Several EMT and stemness markers that are involved in osteomimicry, such as Notch, Met receptor, and Wnt/*β* pathway, were highly expressed in bone metastases.* Conclusions.* Clinical findings support the role of cellular plasticity in PC bone metastasis and suggest that epithelial and mesenchymal states cannot be taken in isolation when targeting PC bone metastasis. The paper also highlights several challenges in the clinical detection of cellular plasticity.

## 1. Introduction

Despite advances in the early diagnosis and management of prostate cancer (PC), bone metastasis of PC cells causes significant morbidity and is associated with four to six times higher mortality rates than localized PC [1, 2]. Furthermore, it is not uncommon for PC patients with bone metastasis who initially respond to androgen deprivation therapy to progress to castration resistant PC (CRPC) [3, 4]. It has been recently recognised that the development of successful therapeutic targets against metastasis is challenged by the presence of a subpopulation of cells within tumours characterised by increased resistance to standard radiotherapy, chemotherapy, and hormonal therapy. These cells, referred to as cancer stem cells (CSCs), have tumour-initiating and self-renewal abilities and are believed to be critical drivers of tumour progression [5].

CSCs have first been described in human leukaemia [6] and subsequently demonstrated in solid tumours, such as those of the breast, brain, and prostate [7–9]. Prostate cancer stem cells have been characterised by a CD44^+^/*α*_2_ *β*_1_^hi^/CD133^+^ phenotype [9]. Importantly, studies have indicated a connection between CSCs and the transition from epithelial to mesenchymal state (EMT) [10–12]. In EMT, polarized immobile epithelial cells convert into spindle-shaped motile mesenchymal cells. This process facilitates detachment of cancer cells from the primary tumour via loss of intercellular adhesion and acquisition of migratory and invasive characteristics. One of the hallmarks of EMT is loss of the transmembrane cell adhesion glycoprotein E-cadherin and an increase in the expression of the mesenchymal markers N-cadherin and vimentin [13, 14]. Several transcription factors have been shown to regulate E-cadherin expression including Snail1, Snail2, Slug, TWIST1, ZEB1, and ZEB2 [13–16]. In addition, maintenance of cellular adhesion between epithelial cells requires the proper interaction between the intracellular domain of E-cadherin and the *β*-catenin protein. Disruption of this E-cadherin/*β*-catenin complex in EMT results in translocation of *β*-catenin into the nuclear compartment and activation of the Wingless (Wnt) signalling pathway [13, 15]. Preclinical models have shown that activation of the Wnt pathway and other signalling pathways such as Notch and transforming growth factor-*β* (TGF*β*) induces EMT and generates stem cell-like phenotypes [17–21].

There is evidence that a transition from a mesenchymal to an epithelial (MET) state, which is the reverse process of EMT, occurs when tumour cells colonise distant sites [22]. The reexpression of E-cadherin facilitates intercellular adhesion between metastatic cells and subsequent tumour growth. This ability of cancer cells to switch between epithelial and mesenchymal phenotypes is referred to as cellular plasticity [23]. An emerging paradigm, based mostly on experimental evidence, proposes that cellular plasticity plays a critical role during the metastatic process, where EMT is critical in the initial invasive and migratory stages, while MET enhances the latter stages of metastatic colonization and growth [22, 24].

The role of cellular plasticity in PC bone metastasis formation is not yet fully understood [25]. In two experimental studies, interaction between the androgen refractory cancer of the prostate (ARCaP) cell lines and bone stromal cells resulted in a mesenchymal phenotype with a switch from E-cadherin to N-cadherin expression [26, 27]. While in another study, similar interactions resulted in an epithelial phenotype with increased expression of E-cadherin [28]. In addition, there have been inconclusive data from experimental studies on the connection between EMT/MET and cancer stem cell states in PC. In one report, pluripotent PC stem cells that express SOX2 and OCT3/4 were found to express E-cadherin [29], while, in two other studies, an EMT phenotype was associated with reduced E-cadherin expression and increased expression of stem cell markers Sox2, Nanog, Oct4, Lin28B, CD44, and/or Notch-1 [12, 30].

These, and other preclinical studies for other types of cancers, suggest that the relation between mesenchymal, epithelial, and CSC states may be more complex than previously viewed and that an epithelial phenotype could promote tumour aggressiveness. For example, breast CSCs have been demonstrated to have the ability to reversibly transition between mesenchymal-like and epithelial-like stem cell states, and it has been shown that the colonization of breast CSCs in bone induces a phenotypic switch from CD44^+^/CD24^−^ to CD44^−^/CD24^+^ cells and co-expression of both epithelial and mesenchymal markers [31, 32]. This ability of CSCs to dynamically transition between epithelial, mesenchymal, and intermediate states facilitates their adaptation to altered microenvironmental stimuli [30]. Hypoxia seems to have a central role in promoting phenotypic transitions and cellular plasticity [33], and E-cadherin has been recently reported to play a role in regulating the response of cancer cells to hypoxia by inducing the expression of hypoxia-inducible factor-1*α* (HIF-1*α*) [34]. These experimental data support a context-dependent role for the epithelial phenotype in enhancing the survival of cancer cells. In addition, E-cadherin has been shown to have a central function in establishing and maintaining the pluripotent and self-renewal properties of prostate CSCs and metastatic tumour-initiating cells [29, 35, 36]. Taken together, these experimental data demonstrate that both epithelial and mesenchymal phenotypes endow metastatic cells with structural and functional properties that allow their survival and growth within the altered environment.

Despite the wealth of* in vitro* and experimental data suggesting the plasticity of cancer cells, it has been difficult to validate cellular plasticity of metastatic tumours in the clinical setting. This is in part because samples taken from patients represent tumour status at a static point in time and do not capture the dynamic phenotypic alterations that have been demonstrated in experimental studies. However, it is imperative that these findings be clinically validated before their translation into potential therapeutic approaches is considered. Therefore, this paper focuses on the clinical evidence of the role of cellular plasticity in PC bone metastasis by reviewing studies that have analysed the differential expression of epithelial, mesenchymal, and stem cell markers in clinical PC bone metastasis tissues compared to primary PC or nonskeletal metastasis tissues.

## 2. Methods

### 2.1. Literature Search and Eligibility Criteria

A search of the PubMed database for English language studies published up to June 2014 was conducted using the following search algorithms for keywords in the title/abstract: (1) (“prostate cancer” OR “prostate carcinoma”) AND (“bone” OR “skeletal” OR “skeleton” OR “osseous”) AND (“metastasis” OR “metastases”) AND (“cadherin” OR “catenin” OR “vimentin” OR “stem” OR “progenitor” OR “tumor initiating” OR “EMT”) and (2) (“prostate cancer” OR “prostate carcinoma”) AND (“bone” OR “skeletal” OR “skeleton” OR “osseous”) AND (“metastasis” OR “metastases”) AND (“tissue” OR “specimen”). Only studies of human bone metastasis tissues were reviewed. Studies based solely on the use of cell lines or animal models, reviews, case reports, letters to editors, and abstracts with no full reports were excluded. The reference lists from included articles were examined to identity further relevant studies.

### 2.2. Data Extraction

The following data were extracted from eligible studies: first author and year of publication, number of tissue specimens analysed and their type, tumour grade or Gleason score, treatment status at time of biopsy, marker(s) studied, method of marker detection, criteria used to score marker expression, differential pattern of expression in bone metastasis tissues, and statistical significance of results. Online supplementary files were used to extract relevant data when they were not available in main article.

### 2.3. Data Synthesis

Descriptive characteristics for studies were summarised. No formal quantitative synthesis (meta-analysis) of the data was performed due to heterogeneity between studies with regard to markers investigated, study population (tumour grade and treatment status), criteria used to score marker expression, and quantitative analysis methods employed.

## 3. Results

### 3.1. Literature Search Results

**Figure 1:**
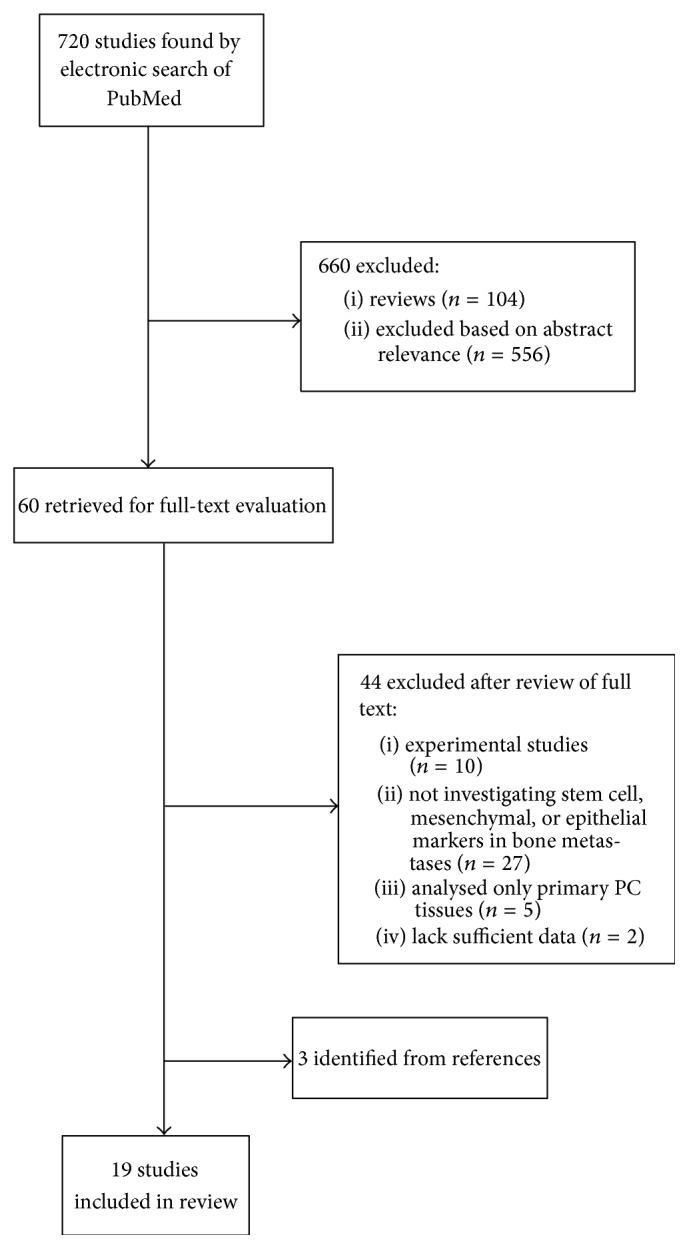
Flow chart of search strategy and study selection.

The electronic search identified a total of 720 studies. Of these, 660 were excluded and 60 studies were retrieved based on abstract relevance. Following full-text assessment, 16 studies were deemed eligible, and three additional relevant studies were identified from reference lists. Thus, a total of 19 studies were included in this review. A flowchart of the search and study selection process is shown in Figure 1.

### 3.2. Study Characteristics

Table 1 summarises the descriptive characteristics of included studies. More than 60% of the studies were published after 2007. Eight studies [37–44] investigated the differential expression of stem cell markers in bone metastasis tissues, and eleven studies [45–55] analysed the differential expression of epithelial markers (mostly E-cadherin) and/or mesenchymal markers (mostly vimentin). Most studies (74%) compared marker expression between primary PC and bone metastasis tissue samples, and only 7 studies used matched tissue samples from the same patients [37, 39, 41, 42, 46, 51, 52].

The median number of bone samples analysed was 17, and around 63% of the studies used ≤20 bone samples. All studies used immunohistochemistry (IHC) for protein detection in bone metastasis samples, except for one study [46], which used* in situ* hybridisation (ISH) for mRNA detection. The study by Armstrong et al. [52] used IHC for marker staining in bone tissues and the FDA-approved CellSearch system for detecting circulating tumour cells. Not all studies reported the tumour grade or Gleason score of the primary or metastasis tissues examined or the treatment status of patients at time of bone biopsy, and among those that did, there was wide heterogeneity in the patient population (details provided in Supplementary Tables 1 and 2) (see Supplementary Material available online at http://dx.doi.org/10.1155/2015/651580).

In general, different criteria were used to score marker expression patterns. Although only two studies did not report scoring criteria, there were variations in the criteria used to categorise positive staining, staining intensity, and staining pattern. Details of these are provided in Supplementary Tables 1 and 2. Eleven studies (58%) reported statistical analysis results of differences in pattern of marker expression between bone metastasis tissues and other tissues, all of which showed statistically significant results. Five out of these eleven studies (45%) used ≤20 bone metastasis specimens.

### 3.3. Expression Pattern of Stem Cell Markers

Table 2 and supplementary Table 1 show details from eight studies that analysed the differential expression of different stem cell markers in bone metastasis tissues compared to primary PC [37, 38, 40–44] and nonskeletal metastasis [38, 39, 43, 44]. The prostate stem cell antigen (PSCA) was found to be overexpressed in bone metastases compared to both primary PC tissues [37] and nonskeletal (lymph node and liver) metastases [39]. C-kit was found to be overexpressed in bone metastases compared to primary PC tissues [40]. A study that analysed the expression of aldehyde dehydrogenase (ALDH) isoforms in 10 PC tissues and their matched bone metastases found positive staining for the ALDH7A1 isoform in both tissues, with absent staining for ALDH1 [42].

Two studies [41, 43] reported different results in relation to the expression of the stem cells markers CD133 and CD44. Castellón et al. [43] found significantly lower expression of both CD133 and CD44 in bone metastases compared to primary tissues. While in the study by Eaton et al. [41], CD44 expression was more frequent in bone metastasis tissues than in primary cancers and CD133 was detected in half of the bone metastases samples. Eaton et al. also found that >70% of bone specimens displayed positive staining for *α*2*β*1 integrin, c-met, and *α*6 integrin, but the staining for CXCR4 was low in both primary and metastases tissues [41]. Sottnik et al. [44] and Knudsen et al. [38] also reported significant overexpression of *α*2*β*1 and Met receptor, respectively, in bone metastases compared to lymph node metastases.

### 3.4. Expression Pattern of EMT/MET Markers

Table 3 and supplementary Table 2 show data extracted from eleven studies that analysed the expression pattern of EMT and MET markers. Only two studies analysed the expression of both epithelial and mesenchymal markers. Sethi et al. [54] found no significant difference in the pattern of expression of E-cadherin or vimentin between primary and bone metastasis tissues. However, in both PC and bone metastasis tissues, reduced expression of E-cadherin was found at the invasive front of the tumour, while it was highly expressed within the tumour centre. Vimentin expression, on the other hand, showed an opposite pattern, where it was overexpressed at the invasive front of the tumour and downexpressed within the tumour centre. In this study, only Notch-1 expression was found to be statistically significantly higher in bone metastasis tissues compared to primary PC [54]. Armstrong et al. [52], on the other hand, found that circulating tumour cells in men with castration resistant prostate cancer (CRPC) coexpress both vimentin and CK (an epithelial marker), while bone metastasis lesions lose vimentin expression and maintain the positive expression of CK. Expression of vimentin was examined in another study [47] and was found to be higher in bone metastasis tissues than in primary PC tissues.

Six studies [45, 46, 49–51, 53] investigated the differential expression of E-cadherin in PC bone metastases. Two of these reported lower expression of E-cadherin in bone metastasis tissues compared to matched primary PC tissues [46, 51]. In contrast, two studies by Saha et al. [49, 50] demonstrated significant upregulation of E-cadherin expression in bone metastases compared to unmatched primary tissue samples, and another study [53] found significantly increased E-cadherin expression in bone compared to soft tissue metastases. Bryden et al. [45] compared different grades of bone metastasis tissues and found an inverse correlation between E-cadherin expression and degree of tumour differentiation, where it was highest in well-differentiated tumours and declined with increasing grade.

Five studies examined changes in the expression of *β*-catenin in bone metastases [46, 48, 49, 51, 55]. Bryden at al. [46] and Pontes et al. [51] found lower *β*-catenin expression in bone metastasis tissues compared to matched primary PC tissues, while Saha et al. [49] demonstrated significantly higher membranous *β*-catenin expression in bone metastases compared to unmatched primary PC samples. It is important to note that in the studies by Pontes et al. [51] and Saha et al. [49], nuclear expression of *β*-catenin (which would indicate EMT) was considered to be negative, while in the study by Bryden et al. [46] the location of *β*-catenin could not be evaluated as mRNA was detected using ISH. Chen et al. [48] reported higher nuclear staining for *β*-catenin and Wnt 1 in bone metastases compared to primary PC and lymph node tissues, with 85% of bone metastases showing strong expression of nuclear *β*-catenin and Wnt 1. In the study by Wan et al. [55], on the other hand, the proportion of bone metastasis tissues that displayed positive nuclear staining for *β*-catenin was lower than that reported by Chen et al. (85% versus 37%). Both studies demonstrated an inverse association between nuclear *β*-catenin expression and androgen receptor status [48, 55].

## 4. Discussion

### 4.1. Summary and Interpretations of Findings

At present, the role of cellular plasticity in bone metastasis formation is not fully clear. Recent preclinical models indicate that the relationship between EMT/MET and CSCs is complex and dynamic. In this paper, we focused on the clinical importance of cellular plasticity in PC bone metastasis by reviewing studies that have comparatively analysed different stemness and EMT/MET markers in bone metastasis and primary tissue samples. We identified methodological limitations and sources of heterogeneity among studies. Sample size in most studies was small, and not all studies used matched primary and metastasis samples from the same patients. In addition, different scoring criteria for defining positive marker expression were used. The study population analysed was heterogeneous with respect to tumour grades and treatment status, which makes direct comparisons between patients difficult. For example, many studies did not report the androgen receptor (AR) status of the tissues analysed. This is important because two studies here showed an inverse association between nuclear *β*-catenin localization and androgen receptor (AR) status, suggesting that reduced AR expression enables Wnt/b-catenin signalling [48, 55]. This is in line with data from the literature that suggests that low AR expression levels are required for EMT in PC cells [56]. Thus, the heterogeneity in expression of EMT and MET markers observed between studies in this review might be partially attributed to differences in AR status in bone metastasis tissues.

Despite these methodological difference between studies reviewed, in general, most stem cell markers analysed, except for CXCR4, were positively expressed in bone metastasis tissues, with an expression level either similar to or higher than primary PC tissues and soft tissue metastases. On the other hand, the expression levels of EMT and MET markers in bone metastasis tissues showed variability within and between samples. This variation does not simply imply that studies are providing conflicting results but highlights the dynamic and transient nature of cellular plasticity that has been reported by preclinical studies and the challenges in capturing this clinically. Pathological samples represent observations of established masses taken at static points in time and are not capable of reflecting the dynamic plasticity of cancer cells. For example, the expression of markers belonging to the metastasis initiating cell population may gradually decrease as foci progress and the metastatic mass becomes more established [41].

**Figure 2:**
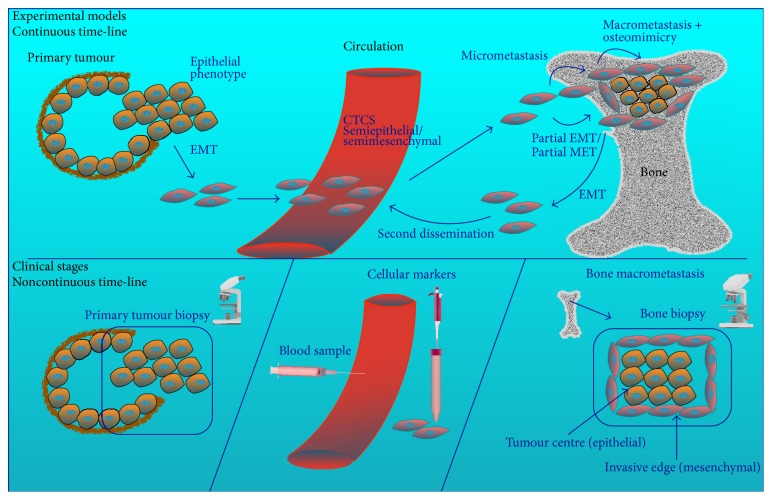
Cellular plasticity of prostate cancer cells over time. The plasticity of cancer cells over time can be delineated from experimental animal and* in vitro* models. Cells in the primary tumour undergo EMT, which enhances their migratory and invasive potential. These invasive cells enter the circulation and may exist as mesenchymal, epithelial, or semimesenchymal/semiepithelial circulating tumour cells (CTCs). Extravasation of CTCs into the bone gives rise to micrometastases. As the metastatic tumour cells colonise the bone and grow into macrometastasis, cells within the tumour centre undergo MET to enable tumour growth and survival under hypoxia, while cells at the invasive edge retain their mesenchymal phenotype to enable invasiveness and osteomimicry. It is suggested that these phenotypic changes are partial and that metastatic cells can dynamically transition between these two states to allow for adaptation to altered microenvironmental stimuli and for further invasion and secondary dissemination. Clinical samples, on the other hand, represent observations of established primary masses and macrometastases at static points in time. Therefore, they cannot capture the dynamic nature of cellular plasticity. Furthermore, current CTC-detection techniques are epithelial-based and cannot capture CTCs that are mostly mesenchymal and have reduced expression of epithelial markers, which would result in missing a significant fraction of CTCs that are predominantly in the mesenchymal state.

Data from studies in this review and others confirm that temporal and spatial factors as well as degree of metastasis differentiation and metastasis size play a critical role in influencing marker expression status at distant sites. The low expression of CXCR4 in bone metastases reported by Eaton et al. [41] suggests that this marker (with its ligand SDF-1) could be important for cancer cell homing to bone and stem cell trafficking [57, 58], but not for distant tumour growth [41]. Thus, as metastases grow, this marker loses its expression. Bryden et al. [45] reported an inverse link between E-cadherin expression and level of tumour differentiation in bone metastases, where E-cadherin expression was highest in well-differentiated tumours and lowest in poorly differentiated ones. Sethi et al. [54] showed evidence for heterogeneity in the expression of EMT and MET markers within bone metastases, where expression of E-cadherin was reduced at the tumour invasive front and was high within the tumour centre, while the expression pattern for vimentin was the opposite. Armstrong et al. [52] provided evidence that circulating tumour cells can exist in an intermediate state expressing both epithelial and mesenchymal markers. In addition, Chao et al. [59] reported an inverse correlation between E-cadherin expression and metastasis size, where E-cadherin expression decreases as tumour mass gets bigger. Taken together, these data suggest that EMT and MET could be partial and reversible and that an increase in epithelial markers in metastasis tissues does not mean a full reversal into an epithelial state. Chao et al. hypothesise that a second EMT can occur at the distant site allowing for further dissemination into other sites [59] (Figure 2).

This plasticity and ability to dynamically transition between epithelial, mesenchymal, and intermediate states facilitates adaptation to altered microenvironmental stimuli [30]. One type of plasticity that allows adaptation in the bone microenvironment is osteomimicry [23]. Several clinical studies in this review found increased expression of markers implicated in osteomimicry. In osteomimicry, PC cells acquire an osteoblast-like phenotype [60] and express bone-related markers, such as osteocalcin, bone sialoprotein [61], osteoprotegerin, and RANKL [62]. Expression of Runx2, a major transcription factor of osteoblast differentiation, is a crucial element in the acquisition of osteomimicry [63]. Several experimental studies demonstrated a positive role for EMT and stemness pathways (e.g., TWIST and Notch) in osteomimicry by promoting the expression of Runx2 [64–66]. In addition, interaction between the hepatocyte growth factor (HGF) and its receptor, Met, has been shown to enhance osteomimicry through activation of the Wnt/*β*-catenin pathway [67, 68]. In this review, positive expression of Met (the stem cell marker and receptor for HGF) in PC bone metastases was demonstrated in two studies [38, 41]; another study reported significant overexpression of the Wnt/nuclear *β*-catenin pathway in bone metastases [48], and Notch expression was found to be significantly higher expression in bone metastasis tissues compared to primary PC tissues [54].

### 4.2. Clinical Challenges and Therapeutic Implications

As noted previously, cellular plasticity is difficult to capture clinically. A clear insight into the dynamics of cellular plasticity in time and space in the clinical context would require comparisons between a large number of matched samples that are serially obtained from primary tumour, circulating cancer cells, bone metastasis, and tumour-associated normal tissues at several points in time and space. This could prove extremely difficult and inconvenient. Furthermore, the currently approved technology for the detection of circulating tumour cells (CTCs) poses some limitations. CellSearch, the only FDA-cleared tool for identifying CTCs relies on the antibodies against the epithelial cell adhesion molecule (EpCAM) for capturing these cells and on the epithelial marker CK for their identification. As this is an epithelial-based detection technique, it cannot capture CTCs that are mostly mesenchymal and have reduced expression of EpCAM. Thus, this method would miss a significant fraction of CTCs that are predominantly in the mesenchymal state [69–71] (Figure 2).

The dynamic phenotypic switching of CSCs in bone metastasis tissues poses important therapeutic implications. The link between EMT and stem cell traits previously reported in the literature has motivated suggestions that counteracting the EMT phenotype could hold promise by targeting cells that are resistant to conventional treatments [25, 70, 72]. However, the evidence for the positive expression of both epithelial and mesenchymal markers in bone metastases implies that therapeutic approaches targeting one of those states might result in an unfavourable activation of the other [73, 74]. Thus, cellular plasticity entails that it may be necessary to target both mesenchymal-like and epithelial-like states in order to eliminate these cells [25]. For example, Bitting et al. [23] propose combining antiepithelial drugs, such as AR antagonists, with drugs against mesenchymal targets such as anti-N-cadherin antibodies in the treatment against metastasis.

Targeting the cancer stem cell phenotype might prove to be better than targeting epithelial and mesenchymal states. Emerging insights into the role of microRNAs in cellular plasticity could provide novel therapeutic approaches. MicroRNAs are small noncoding RNAs that mediate posttranscriptional gene regulation. These molecules have been implicated in the network regulating cellular plasticity and stemness in PC progression [75, 76]. For example, Liu et al. [75] demonstrated that microRNA-34a could be a potential therapeutic target against PC stems cells. In addition, microRNA-143 and microRNA-145 have been shown by three studies [77–79] to potentially play a role in PC bone metastasis by targeting the EMT regulator HEF1 [79] and the stem cell markers CD133, CD44, Oct-4, c-myc, and Klf4 [78]. Nevertheless, the microRNA-detection methods employed in these studies were not cell type-specific, and it is yet to be determined whether these microRNAs were expressed by tumour cells or by the surrounding bone stroma [80].

In conclusion, although findings suggest a potential role of CSCs in PC bone metastasis and stemness as a mediator of cellular plasticity, there are major challenges in detecting cellular plasticity in the clinical setting and questions remain unanswered as to whether phenotypic transitions are full or partial, whether nonstem cancer cells undergo phenotypic switching as well, and when and how do these phenotypic transitions occur.

## Supplementary Material

The two tables in the supplementary material contain additional details about the 19 studies reviewed. These details pertain to the Gleason score of the primary tumour, the treatment status at the time of bone biopsy, tissue type (e.g. formalin fixed, paraffin embedded, etc), marker detection method used (Immunohistochemistry or In Situ Hybridisation), and the scoring criteria used for defining positive marker expression. Supplementary table 1 contains details for the 8 studies that investigated the differential expression of stem cell markers, and Supplementary table 2 contains details for the 11 studies that analysed the differential expression of EMT/MET markers.

## Figures and Tables

**Table 1:** Descriptive characteristics of included studies.

Year of publication	Number of studies

1999	1
2000	1
2002	3
2004	1
2005	1
2008	3
2010	3
2011	3
2012	2
2013	1

Sample size	Median (range)

Number of BM specimens	17 (2–184)
Number of primary PC specimens	22 (6–112)
Number of nonskeletal specimens	23 (7–97)

Tissue comparisons	Number of studies

Studies that used matched samples	7
Comparisons between BM and primary PC	14
Comparisons between BM and nonskeletal metastases	7
Comparisons between different BM tissues	2

Markers analysed	Number of studies

Stem cell markers	8
EMT/MET markers	11

Main method of marker detection	Number of studies

Immunohistochemistry (IHC)	18
In situ hybridisation (ISH)	1

Tumour grade and treatment status	Number of studies

Studies that reported tumour grade	12
Studies that reported treatment status	9

Criteria used to score expression	Number of studies

Pattern of distribution of staining across cell	6
Percentage of positive cells in each specimen	5
Staining intensity scoring	2
Staining intensity multiplied by % of positive cells	5
Quantitative analysis of % of positive staining areas using Image Pro Plus 6.2 software	1
Did not report a scoring method	2

Reporting of results	Number of studies

Statistical analysis of differential expression	11

Note: BM: bone metastasis.

**Table 2:** Details of studies analysing expression of stem cell markers in PC bone metastases.

Study	Marker	No. of BM specimens	Comparison tissue (No. of specimens)	Results	Significance
Gu et al., 2000 [37]	PSCA	9	Normal (25) Primary PC (112, 3 matched)	All normal tissues were negative. PSCA expression was positive in 105/112 (94%) primary and in 9/9 (100%) BM	NR

Knudsen et al., 2002 [38]	Met receptor	45	Primary PC (90) LN (35) Other soft tissues (8)	High Met expression in 52% of primary, 83% of BM, and 54% of LN metastases	*P* = 0.0003 for BM versus LN

Lam et al., 2005 [39]	PSCA	47	LN (6, 5 matched) Matched liver (3)	PSCA staining intensity was higher in BM (87%) compared with LN (67%) and liver (67%) metastases	*P* = 0.014 for BM versus LN. No statistical analysis for liver metastases due to small sample size

Wiesner et al., 2008 [40]	c-kit, SCF	20	Primary PC (21) BPH (22)	Positive staining for c-kit in 5% of BPH, 14% of primary PC, and 40% of BM Positive SCF staining in 95% of BPH and primary PC and in 85% of BM specimens	*P* = 0.0077 for BM versus BPH

Eaton et al., 2010 [41]	CD133, CD44, *α*2*β*1 integrin, CXCR4, c-met, *α*6 integrin	11	Matched primary PC (11)	50% of samples positive for CD133. >70% positive for CD44, *α*2*β*1, c-met, and *α*6 integrin. No difference in expression between primary and BM samples for all markers except for CD44. Higher expression of CD44 in BM, with positive staining in bone stoma. Low staining for CXCR4 in both BM and primary samples	NR

van den Hoogen et al., 2010 [42]	ALDH isoforms	10	Matched primary PC (10)	No staining for ALDH1 in BM or primary PC ALDH7A1 in 7/10 primary PC and 8/10 BM with no staining in bone stroma	NR

Castellón et al., 2012 [43]	CD133, CD44	5	Primary PC (34) LN (7)	BM and LN showed lower expression of both CD133 and CD44 compared with primary tissues	*P* < 0.05 for BM and LN versus medium Gleason grade

Sottnik et al., 2013 [44]	*α*2*β*1	184	BPH (43) Primary PC (76) LN (61) Liver (36)	Higher expression in BM compared to primary PC and nonskeletal metastases	*P* < 0.042 for BM versus LN

ALDH: aldehyde dehydrogenase, BM: bone metastasis, BPH: benign prostatic hyperplasia, LN: lymph node, No.: number, NR: not reported, PSCA: prostate stem cell antigen, and SCF: stem cell factor.

**Table 3:** Details of studies analysing expression of EMT/MET markers in PC bone metastases.

Study	Marker	No. of BM specimens	Comparison tissue (No. of specimens)	Results	Significance for differential expression
Bryden et al., 1999 [45]	E-Cadherin	20	Different grades of BM tissues	Decrease in expression with increasing tumour grade	NR

Bryden et al., 2002 [46]	E-Cadherin, *β*-catenin	14	Matched primary PC (14)	mRNA for both markers was expressed uniformly in 9/14 primary PC. In BM, 6/14 showed negative mRNA for one or both markers and 6/14 expressed both heterogeneously	NR

Lang et al., 2002 [47]	Vimentin	8	Primary (54)	16/54 of primary expressed vimentin with ≤50% staining cells. 7/8 BM expressed vimentin with 100% of cells staining positive in 5 specimens	NR

Chen et al., 2004 [48]	Wnt-1/*β*-catenin	23 (from 9 patients)	Primary PC (62, from 49 patients) LN (23, from 9 patients)	High Wnt-1/*β*-catenin in 85% of BM and 77% of LN. Highest expression was observed in BM	*P* < 0.05 for BM (& for LN) versus normal tissues

Saha et al., 2008 [49]	E-Cadherin, *β*-catenin	17	Primary PC (22) BPH (11)	BM tissues showed higher frequency of homogenous expression of both markers compared to primary PC	*P* < 0.001 for BM versus primary PC

Saha et al., 2008 [50]	E-Cadherin	15	Primary PC (20) BPH (11)	Higher expression of unmethylated gene and homogenous protein in BM compared to primary tissues	*P* < 0.001 for BM versus primary PC

Pontes et al., 2010 [51]	E-Cadherin, *β*-catenin	28	Matched primary PC (6)	For E-cadherin & *β*-catenin, respectively, there was abnormal expression in 86% and 82% of 28 BM specimens. In matched specimens, normal expression was seen in 5/6 and 3/6 of primary PC and abnormal expression was seen in 5/6 of BM for both markers	NR

Armstrong et al., 2011 [52]	Vimentin, CK	2	CTCs (10, 2 matched)	Coexpression of vimentin and CK in 10/10 of CTCs Absent vimentin expression in 2/2 of CK-positive BM. Strong vimentin expression in bone stroma	NR

Putzke et al., 2011 [53]	E-Cadherin	109	LN (30) Liver (18) Other (8)	Higher expression in BM compared to all soft tissue metastases	*P* < 0.01 for BM versus all soft tissue metastases

Sethi et al., 2011 [54]	E-Cadherin, Vimentin, PDGF-D, NF-kB, Notch-1, ZEB1	10	Primary PC (10)	Higher expression of Notch-1 in BM compared to primary PC. No quantitative difference in expression for other markers. In primary and BM, EMT markers (vimentin & NF-kB) had higher expression at invasive tumour front than within tumour centre. The opposite pattern was observed for E-cadherin	For BM versus primary PC: *P* = 1 for E-cadherin, vimentin, PDGF-D, NF-kB, *P* = 0.057 for Notch-1, NR for ZEB1

Wan et al., 2012 [55]	*β*-catenin	27	Association with AR expression in BM tissues	Nuclear localization of *β*-catenin in 10/27 BM specimens. Localization was inversely associated with AR expression	*P* = 0.056 for inverse association between nuclear localization of *β*-catenin and AR expression

AR: androgen receptor, BM: bone metastasis, BPH: benign prostatic hyperplasia, CK: cytokeratin, CTCs circulating tumour cells, ISH: *in situ* hybridisation, LN: lymph node, MS-PCR: methylation specific polymerase chain reaction, No.: number, and NR: not reported.
