# Supplementary material for: Cellular Plasticity in Prostate Cancer Bone Metastasis
Source: Prostate Cancer. 2015 Jun 3;2015:651580. doi: 10.1155/2015/651580 (PMC4469842; doi:10.1155/2015/651580)
Supplement: Supplementary file 1 — The two tables in the supplementary material contain additional details about the 19 studies reviewed. These details pertain to the Gleason score of the primary tumour, the treatment status at the time of bone biopsy, tissue type (e.g. formalin fixed, paraffin embedded, etc), marker detection method used (Immunohistochemistry or In Situ Hybridisation), and the scoring criteria used for defining positive marker expression. Supplementary table 1 contains details for the 8 studies that investigated the differential expression of stem cell markers, and Supplementary table 2 contains details for the 11 studies that analysed the differential expression of EMT/MET markers. [file 651580.f1.pdf]

Supplementary Table 1: Additional Details for Stem-cell Marker Studies

Study	Primary PC GS (No. of Specimens)	Treatment Status	Tissue Type	Marker Detection Method	Expression Scoring Criteria
Gu et al., 2000 [37]	2-4 (4)<br>5-6 (27)<br>7 (47)<br>8-10 (34)	6 patients treated prior to surgery with hormone ablation therapy	Paraffin-embedded	IHC	Score of 0-9 based on % of positive cells (0=0%, 1=25%, 2=25-50%, 3≥50%) & staining intensity (0=0, 1=1+, 2=2+, 3=3+).
Knudsen et al., 2002 [38]	7-6 (90)	No patient received neoadjuvant hormonal or postoperative therapy	TMA for metastases	IHC	Staining rated as low (1+), moderate (2+), or high (3+) based on intensity score (1-3) multiplied by % of positive cells.
Lam et al., 2005 [39]	NR	Patients received anti-androgen therapy and/or chemotherapy	Formalin-fixed, paraffin-embedded	IHC	Staining intensity graded as 0 (no staining), 1+ (weak staining), 2+ (moderately intense staining), or 3+ (severely intense staining). Staining density quantified as % of positive staining cells.
Wiesner et al., 2008 [40]	5-6 (9)<br>7+ (12)	NR	Formalin-fixed, paraffin-embedded	IHC	NR
Eaton et al., 2010 [41]	NR	All patients untreated at time of bone biopsy	Bone specimens were fixed in buffered formaldehyde & demineralized over 3 weeks in EDTA	IHC	Staining pattern (membranous, punctate, or cytoplasmic) and<br>% of positive stained cells.
van den Hoogen et al., 2010 [42]	NR	NR	TMA	IHC	Staining score reported for ALDH7A1 (0: no staining, 1: moderate staining, 2: strong staining)
Castellón et al., 2012 [43]	Low Gleason grade 2 (11)<br>Medium Gleason grade 3 (14)<br>High Gleason grade 4-5 (9)	NR	Formalin-fixed, paraffin-embedded	IHC	Quantitative analysis of % of positive staining area using Image ProPlus 6.2 software.

Sottnik et al., 2013 [44]	NR	NR	TMA	IHC	Staining intensity graded as 0 (absent, no staining), 1 (weak, faint or fine chromogen deposition), or 2 (strong, clear & coarse granular chromogen deposition), multiplied by % of positively stained cells.

IHC Immunohistochemistry, NR Not Reported, TMA Tissue Microarray

Supplementary Table 2: Additional Details for EMT/MET Marker Studies

Study	Primary PC GS (No. of Specimens)	Treatment Status	Tissue Type	Detection Method	Expression Scoring Criteria
Bryden et al., 1999 [45]	Grades for BM: Well-differentiated (3) Moderately-differentiated (4) Poorly-differentiated (3)	All patients untreated at time of bone biopsy	Formalin-fixed, wax-embedded	IHC	Semi-quantitative grading based % of positively stained cells. Non-membranous expression of E-cadherin graded negative
Bryden et al., 2002 [46]	5-7 (3) 8-10 (11) All BM were poorly differentiated	All patients untreated at time of bone biopsy	Formalin-fixed, paraffin-embedded. BM decalcified in EDTA	ISH	Distribution of mRNA signal using ISH across cell graded as uniform, heterogeneous or negative.
Lang et al., 2002 [47]	2-4 (15) 5-7 (14) 8-10 (25)	Patients untreated at time of bone biopsy	Formalin-fixed, paraffin-embedded	IHC	Scores based on % of vimentin positive cells within a tumour (0=no reactive cells, 1=< 25%, 2= 26-50%, 3=51-75%, 4=76-99%, 5=100%).
Chen et al., 2004 [48]	≤7 (30) >7 (18)	NR	Paraffin-embedded	IHC	Brown membrane and cytoplasmic staining for Wnt-1, diffusive cytoplasmic and nuclear staining for β-catenin, were scored as positive.
Saha et al., 2008 [49]	8-10 (22)	NR	Formalin-fixed, paraffin-embedded	IHC	Staining pattern (uniform, heterogeneous, or negative).

Saha et al., 2008 [50]	5-7 (3)<br>8-10 (17)	NR	Formalin-fixed, paraffin-embedded	IHC	Staining pattern (uniform, heterogeneous, or negative) and E-cadherin gene methylation using MS-PCR
Pontes et al., 2010 [51]	6 (1)<br>7 (2)<br>8 (1)<br>9 (2)	None of the 6 patients with matched specimens received radiotherapy or chemotherapy. Of the rest: 8 patients were on androgen-deprivation therapy & treatment status was unknown for 9 patients.	Formalin-fixed, paraffin-embedded. BM were decalcified with 7% nitric acid	IHC	Graded as normal (>70% positive membranous staining) or abnormal (weak, focal, or nuclear staining)
Armstrong et al., 2011 [52]	For a total of 41 men with mCRPC, median GS was 8 (range: 5-10)	For a total of 41 men with mCRPC, 68% has prior chemotherapy, 73% prior bisphosphonates, and median No. of hormonal therapies (range) was 2.5 (0-5).	Formalin-fixed, paraffin-embedded	IHC for BM CellSearchsystem for CTCs	NR
Putzke et al., 2011 [53]	NR	NR	TMA	IHC	Staining intensity score (0-2 for nuclear, cytoplasmic & membranous) multiplied by % of positive cells.
Sethi et al., 2011 [54]	NR	NR	Formalin-fixed, paraffin-embedded. BM decalcified in EDTA	IHC	Staining intensity score (0-3 for negative, weak, medium & strong) multiplied by % of stained cells score (1-3).
Wan et al., 2012 [55]	NR	NR	Formalin-fixed, paraffin-embedded, decalcified in formic acid	IHC	For β-catenin, scoring based on % of cells with positive nuclear, cytoplasmic & membranous staining. For AR, scores based on % of cells with positive nuclear staining.

Abbreviations: BM Bone Metastasis, CRPC Castration Resistant Prostate Cancer, CTCs Circulating Tumour Cells, GS Gleason Score, IHC Immunohistochemistry, ISH In Situ Hybridisation, mCRPC metastatic Castration Resistant Prostate Cancer, NR Not Reported, TMA Tissue Microarray
